# Dealing with sickness certification – a survey of problems and strategies among general practitioners and orthopaedic surgeons

**DOI:** 10.1186/1471-2458-7-273

**Published:** 2007-10-02

**Authors:** Britt Arrelöv, Kristina Alexanderson, Jan Hagberg, Anna Löfgren, Gunnar Nilsson, Sari Ponzer

**Affiliations:** 1 Department of Development in Health and Medical Services, FORUM, Box 175 33 Stockholm, Sweden; 2 Section of Personal Injury Prevention, Department of Clinical Neuroscience, Karolinska Institutet, Stockholm, Sweden; 3 Department of Family medicine, Karolinska Institutet, Stockholm, Sweden; 4 Department of Clinical Science and Education, Södersjukhuset, Karolinska Institutet, Stockholm, Sweden

## Abstract

**Background:**

In order to get sickness benefit a sick-listed person need a medical certificate issued by a physician; in Sweden after one week of self-certification. Physicians experience sick-listing tasks as problematic and conflicts may arise when patients regard themselves unable to work due to complaints that are hard to objectively verify for the physician. Most GPs and orthopaedic surgeons (OS) deal regularly with sick-listing issues in their daily practice. The aim of this study was to explore perceived problems and coping strategies related to tasks of sickness certification among general practitioners (GP) and orthopaedic surgeons (OS).

**Methods:**

A cross-sectional study about sickness certification in two Swedish counties, with 673 participating GPs and 149 OSs, who answered a comprehensive questionnaire. Frequencies together with crude and adjusted (gender and working years) Odds ratios were calculated.

**Results:**

A majority of the GPs and OSs experienced problems in sickness certification every week. To assess the patient's work ability, to handle situations when they and the patient had different opinions about the need for sickness absence, and to issue prolongation certificates when the previous was issued by another physician were reported as problematic by a majority in both groups. Both GPs and OSs prolonged sickness certifications due to waiting times in health care or at Social Insurance Office (SIO). To handle experienced problems they used different strategies; OSs issued sickness certificates without personal appointment more often than the GPs, who on the other hand reported having contact with SIO more often than the OSs. A higher rate of GPs experienced support from management and had a common strategy for handling sickness certification at the clinic than the OSs.

**Conclusion:**

Most GPs and OSs handled sickness certification weekly and reported a variety of problems in relation to this task, generally GPs to a higher extent, and they used different coping strategies to handle the problems.

## Background

Most sickness insurance systems in the Western countries require that patients present a medical certificate issued by a physician after some days of self certification (in Sweden seven days), in order to get sickness benefits when they are unable to work due to disease or injury. All physicians in Sweden are entitled to issue sick-listing certificates.

Earlier studies have shown that physicians experience such tasks as problematic [1]. For instance, sick-listing activities might result in conflicts with patients and loyalty dilemmas [1-7], and are considered to have a substantial impact on at least general practitioners' (GPs') overall workload [8,9]. Prominent differences in experience with sickness certification among GPs have also been shown [10]. Problems occur and conflicts may arise when patients regard themselves unable to work due to complaints that are hard to objectively verify for the physician [11-14]. Frequently the physician has to rely on the patient's story and evaluation of his or her ability to work. The role of physicians in the sick-listing process has been discussed, especially when the absence rate increase [2,3,12,15,16].

Most sickness absence in Sweden is due to musculoskeletal or psychiatric disorders [1]. Consequently, most GPs and orthopaedic surgeons (OS) deal regularly with sick-listing issues in their daily practice. Most previous studies in sickness certification practices have focused on GPs [1,2,7,9-11,13,17-23], while there are few studies including also OSs and other physician categories [24-28]. The few published studies show differences in sickness certification practices between different physicians and physician categories. GPs and OSs have been found to have different attitudes towards sickness certification and to assess work inability differently [27]. However, so far the number of scientific studies on this is very limited and mainly has used small and selected populations [1]. As mentioned, it is evident that the sick-listing are considered problematic, but knowledge is needed on a more detailed level about specific problems and on the related personal and organisational approaches for coping with them, to design adequate interventions. GPs and OSs are two groups of physicians that generally have such cases at a daily bases, but are working in different contexts and thereby have different possibilities to deal with problematic sick-listing issues.

The aim of this study was to explore perceived problems, and coping strategies related to sickness certification among GPs and OSs. In the article we report and discuss our findings.

## Methods

### Design of the study

A comprehensive questionnaire, with 83 fixed response questions about perceived problems, proficiency regarding sick-listing together with questions about cooperation with Social Insurance Office and need of training, was used. The questions were based on earlier knowledge from the literature and interviews with physicians.

### Setting

About 7700 physicians below the age of 65 in two counties in Sweden (Stockholm and Östergötland) were contacted by mail and asked to participate in the study. In the Stockholm County the register of the members of the Swedish Medical Association was used to identify physicians working in Stockholm. Since 95% of all physicians are members of the Swedish Medical Association virtually all physicians were reached. In Östergötland a register used for advertising purposes was used (Pharma Marketing AB) including 100 % of all physicians. The questionnaires including two reminders were managed by Statistics Sweden. The study was approved by the Regional Ethical Review Board in Stockholm.

### Participants

A total of 5455 physicians (71.2 %), answered the questionnaire [29]. In this study the answers from the following two groups of physicians were analysed; the physicians who handled sick-listing tasks at least a few times per year, were specialists, and worked in a GP office (n = 673) or in an orthopaedic surgery (n = 149), Table 1. In Sweden the specialist training for both GP and OS is about five years.

**Table 1:** Comparison between orthopaedic surgeons and general practitioners regarding background data

	Total	Orthopaedic surgeons			General practitioners		
	n	n	mean/%	SD	n	mean/%	SD

Age, mean	822	149	51.1	7.08	673	52.2	6,70
Working years, mean	797	140	22.3	7.60	657	20.6	7.30
Sex, men %	822	127	85.2		322	47.8	

### Statistical analysis

The questions and response alternatives are listed in respective Table 2, 3, 4, 5. Based on type of questions we have chosen to define them as dealing with personal stressors, personal strategies, roles etcetera, as seen in the tables. Descriptive statistics including estimation of p-values from chi-2 tests were calculated using SPSS. Two logistic regressions were performed for questions dichotomised to binary variables. In Tables 2 and 4 the variables were dichotomised to every week versus less often, in Table 3 to very or rather problematic versus hardly problematic or not problematic, and in Table 5 to yes and partly versus no. In the simple regressions, workplace was used as the independent variable and crude odds ratios (OR) including 95% confidence intervals (CI) were reported. In the multiple regressions, gender and working years were used as independent variables when calculating adjusted ORs for workplace including 95% CI. There were missing values for 25 of a total of 822 physicians regarding working years and no missing values for the other variables. Missing value statistics in the 27 simple regressions were: minimum = 0, median = 5, maximum = 18. The corresponding statistics for the multiple regressions were: minimum = 25, median = 29, maximum = 42. In response to the question on threats in Table 3, only one orthopaedic surgeon reported experiencing threats weekly. There were no other problems with few counts in the cells.

**Table 2:** General problems, personal stressors perceived, and strategies used regarding sickness certification

How often do you.......	Orthopaedic surgeons				General practitioners					Odds ratio^3^			
	
	weekly^1 ^%	monthly %	yearly %	never %	weekly %	monthly %	yearly %	never %	P^2^	crude	95%CI	adjusted^4^	95%CI
..have consultations that include consideration of sickness certification	97.3	2.7	0.0	0.0	97.1	2.2	0.7	0.0	Ns	0.9	0.3–2.7	1.0	0.3–3.6
..find it problematic to handle sickness certification	53.0	26.5	12.9	7.5	61.4	31.3	6.0	1.4	S	1.4	0.3–2.0	1.4	0.9–2.0
*Personal stressors*													
..encounter a patient who wants to be on sick leave for some reason other than work incapacity due to disease or injury	26.3	33.1	24.3	16.2	35.3	39.8	19.7	5.2	S	1.5	1.0–2.3	1.6	1.0–2.5
.. have conflicts with patients about sickness certification	13.6	30.6	39.5	16.3	19.9	32.2	39.0	8.8	S	1.5	1.0–2.6	1.5	0.9–2.5
..feel threatened by a patient in connection with sickness certification	0.7	2.0	16.1	81.2	2.2	5.5	25.0	66.0	s	5.3	0.7–39.5	4.6	0.6–35.1
*Personal strategies*													
..have contact with SIO^5 ^staff about matters concerning sickness certification	10.7	28.9	42.3	18.1	23.4	50.7	23.2	2.7	s	4.4	3.0–6.3	4.2	2.8–6.2
.. make a referral to occupational health	10.0	30.9	32.2	26.8	10.2	36.5	38.9	14.2	s	1.0	0.6–1.9	0.9	0.5–1.8
..issue sickness certificates without personal appointment	36.5	23.0	16.2	24.3	18.3	33.2	22.0	26.3	s	0.4	0.3–0.6	0.5	0.3–0.7

^1 ^Following response alternatives are combined; more than 20 times a week, 6–20 times a week, 1–5 times a week

^2 ^The significance of difference between physician categories on the 5% level (Chi2 test with four response alternatives). s = significant, ns = non-significant

^3 ^The Odds ratio for dichotomized (weekly- less than weekly) response alternatives. Reference group; OS = 1

^4 ^Adjusted for gender and working years

^5 ^SIO = Social insurance office

**Table 3:** Physicians reporting "very problematic" or "rather problematic" about tasks related to sickness certification

	OS	GP		Odds ratio^3^			
Type of problem^1^	%	%	P^2^	crude	95% CI	adjusted^4^	95% CI
*Assessment*							
To assess whether a patient's functional capacity is reduced	19.4	63.6	s	7.2	4.7–11–2	7.1	4.5–11.3
To assess the degree to which reduced functional capacity limits a patient's work ability	53.4	80.9	s	3.7	2.5–5.4	4.0	2.7–6.1
To ascertain the optimum duration and degree of sickness certification	44.3	77.6	s	4.4	3.0–6.3	4.2	2.8–6.3
To suggest a plan of action and/or measures to be taken during the sick leave?	37.8	55.2	s	2.0	1.4–2.9	2.1	1.4–3.0
*Certification*							
To issue sickness certificates to be used by SIO^5^	47.0	46.4	ns	1.0	0.7–1.4	0.9	0.6–1.3
To decide whether to authorize prolongations of a period of sick leave that was previously certified by another physician?	52.0	75.1	s	2.8	1.9–4.0	2.4	1.6–3.5
*Role*							
To manage the two different roles as the patient's physician and medical expert for the social insurance offices and other authorities?	39.9	65.1	s	2.9	2.0–4.2	2.9	1.9–4.4
To handle situations in which you and a patient have different opinions about the need for sickness absence?	52.4	81.0	s	3.9	2.7–5.7	3.5	2.3–5.2
To discuss with patients the advantages and disadvantages of being on sick leave?	30.2	50.7	s	2.4	1.6–3.5	2.2	1.4–3.2

^1 ^The questions began "How problematic do you find it ... and the response alternatives were; very problematic, fairly problematic, not very problematic, not problematic

^2 ^The significance of difference between physician categories on the 5% level (Chi2 test with four response alternatives). s = significant, ns = non-significant

^3 ^Odds ratio dichotomized (problematic-not problematic) response alternatives. Reference group; OS = 1

^4 ^Adjusted for gender and working years

^5 ^SIO = Social insurance office

**Table 4:** Prolonged sickness certification due to waiting times and deficient treatment opportunities

	Orthopaedic surgeons				General practitioners^1^				Odds ratio^3^			
	weekly^2 ^%	monthly %	yearly %	never %	weekly %	monthly %	yearly %	never %	crude	95% CI	adjusted^4^	95% CI

*Within health care*												
Waiting times for investigation	37.2	25.7	18.2	18.9	29.0	39.8	23.7	7.5	0.7	0.5–1.0	0.7	0.5–1.1
Waiting time for treatment	42.2	27.2	19.7	10.9	28.9	36.1	29.3	5.7	0.6	0.4–0.8	0.6	0.4–0.9
Lack of access to adequate care/care providers	5.4	9.5	19.6	65.5	20.4	35.9	31.5	12.1	4.5	2.2–9.4	5.4	2.3–12.7
*Outside health care*												
Waiting times at Social Insurance Office	19.6	23.6	27.7	29.1	30.1	38.4	24.2	7.4	1.8	1.1–2.7	1.8	1.1–2.8
Wait for measures taken by the employer	6.9	17.9	31.7	43.4	14.5	31.8	32.4	21.3	2.3	1.2–4.5	2.6	1.2–5.6
Waiting times at unemployment office	7.5	18.9	31.8	41.9	12.5	28.1	34.7	24.6	1.8	0.9–3.4	2.0	0.9–4.1

^1 ^All differences between the physician categories were significant at the 5% level (Chi2 test with four response alternatives)

^2 ^The following response alternatives are combined; more than 20 times a week, 6–20 times a week, 1–5 times a week

^3 ^The Odds ratio for dichotomized (weekly vs. less than weekly) response alternatives. Reference group; OS = 1

^4 ^Adjusted for gender and working years

**Table 5:** Support from management and common strategies at clinic regarding sick-listing matters

	Orthopaedic surgeons				General practitioners				Odds ratio^1^			
	n	yes %	partly %	no %	n	yes %	partly %	no %	crude	95% CI	adjusted^2^	95% CI

Having support from management regarding handling of sickness certification cases	141	26.2	32.6	41.1	663	32.1	42.5	25.3	2.1	1.4–3.0	2.1	1.4–3.1
*Physicians reporting problems every week^3^*	*75*	*28.0*	*37.3*	*34.7*	*404*	*30.2*	*43.6*	*26.2*				
*A *common strategy for handling matters related to sickness certification at the clinic/practice	144	9.7	35.4	54.9	673	15.9	53.9	30.2	2.8	1.9–3.2	2.1	1.1–3.9
*Physicians reporting problems every week ^4^*	*75*	*9.3*	*36.0*	*54.7*	*408*	*14.0*	*55.1*	*30.9*				

^1 ^The Odds ratio for dichotomized response alternatives (yes and partly vs. no). Reference group; OS = 1

^2 ^Adjusted for gender and working years

^3 ^A subgroup of physicians answering the question "Having support from management regarding handling of sickness certification cases" reporting that they every week "Find it problematic to handle sickness certification"

^4 ^A subgroup of physicians answering the question "*A *common strategy for handling matters related to sickness certification at the clinic/practice" reporting that they every week "Find it problematic to handle sickness certification"

## Results

There were no significant differences between the GPs (n = 673) and the OSs (n = 149) regarding age, however, OSs had worked as physicians significantly longer than the GPs and there was a significantly higher rate women among the GPs, Table 1.

### General problems, personal stressors, and strategies

In both groups of specialists, 97% met sick-listing patients every week, Table 2, and a majority experienced problems weekly with sickness certification. At a more detailed level, most physicians experienced problems with "to encounter a patient who wants to be on sick leave for some reason other than work incapacity due to disease or injury". A larger rate of GPs reported personal stressor. Among GPs the most frequent personal strategy used was "to have contacts with social insurance staff...", and for OSs it was "to issue sickness certificates without a personal appointment". All differences between the two groups were statistically significant.

### Clinical problems

The most common types of clinical problems were "to assess the degree to which reduced functional capacity limits a patient's work ability", "to handle situations in which you and a patient have different opinions about the need for sickness absence", and "to decide whether to authorize prolongations of a period of sick leave that was previously certified by another physician", Table 3. All factors studied except for "to issue sickness certificates to be used by Social Insurance Offices (SIO)" were experienced as significantly more problematic by the GPs than by the OSs. Although the ORs decreased somewhat for most of the questions, the differences did not significantly change when adjusting for gender and working years.

### Sickness absence due to waiting times

A higher proportion of OSs issued prolonged sick-listings every week more than would have been necessary due to waiting time for investigation and treatment in health care as compared to GPs, Table 4. On the other hand, more GPs prolonged sickness certificates of medical reasons, due to that their patients had to wait for measures to be taken by the SIO or the employer. Furthermore, they had a four times higher OR for issuing certificates when they had not enough access to adequate care or care providers for the patient.

### Organisational strategies

"Support from management regarding handling of sickness certification cases" was experienced by 32% of GPs, and 26% of OSs, while 30% of the former and 55% of the latter had no "common strategy for handling matters related to sickness certification at the clinic", Table 5. The figures did not change much when only considering those who had reported frequent (i.e. every week) problems in handling sickness certification. Nor did the ORs change when adjusting for sex and working years.

## Discussion

Our study reveals that both GPs and OSs frequently experience a variety of problems with sickness certification, with GPs experiencing the most personal stressors and clinical problems, and that personal strategies in order to cope with them differed widely between the specialist groups.

The results emphasise that different physician categories meet different kinds of patients in different types of contexts, leading to varying possibilities for handling duties such as sickness absence certification. OSs and GPs represent two categories of physicians in the Swedish health care system that regularly deal with sickness absence certification issues without this being their main task. There were more men in the OS group and they had at average worked as physicians for more years than the GPs. These facts, however, did not have a significant influence on the results. Some earlier studies have shown differences between physicians of different genders while others have not [1,23,24,27]. In this study we found that type of speciality rather than gender or working years was much more important for the results.

### Strengths and limitations

Strengths of this study is that physicians working in the two participating counties represent one fifth of all physicians in Sweden, that practically all GPs and OSs in the area were included, and the high response rate (71%). Social Insurance legislation and its administration is the same throughout Sweden as is the sick-listing role of GPs and OSs. The results might, thereby, be considered representative for most Swedish GPs and OSs. A limitation is that the dropout rate could not be related to specialities. This was the first time the questionnaire was used, except for a smaller pilot study. The reliability of the instrument was not tested. Based on how the questionnaire was developed, and on comments from participants, other physicians, as well as researchers in the area, face validity can be claimed. So far, other validity tests have not been made.

### Personal stressors and strategies

A vast majority of both GPs and OSs stated that they found at a weekly basis handling sickness certification matters as problematic. The experience varied widely between the two groups of doctors. However, experience has earlier been shown to vary between different groups of GPs [23]. One important factor explaining why GPs reported more problems than the OSs, might be that the GPs more frequently encountered patients who wanted to be on sick leave for some reason other than work incapacity due to disease or injury. This might in itself also explain why the GPs experienced more conflicts with and threats from their patients. On the other hand, they had more regular contacts with social insurance officers and referred patients somewhat more often to occupational health services, implying that they might be able to get more support in these issues. OSs on the other hand issued more certificates without a personal appointment with the patient.

Better cooperation and more frequent contact between health care and SIO have been suggested as one important solution for sickness absence problems in health care [8]. Despite the fact that more of the GPs reported having regular planned contacts with the SIO, they perceived many more problems than the OSs. The SIO has, thus far, offered educational activities to GPs to a much greater extent than to other physician categories. Perhaps these contacts and educational activities have not been adapted to the problems perceived by the GPs, but have instead made them more aware of problems related to sickness absence certification as compared to the OSs. GPs also seem to have less opportunity to use administrative measures in coping with sickness certifications.

### Clinical problems

Most of the OSs did not find it problematic to assess the patient's functional capacity, in contrast to the GPs, who found it much more problematic. One reason for this difference might be that the two categories of physicians met different types of patients, and many of the OSs' patients had been referred to them by other physicians (i.e. GPs or occupational health specialists). However, assessing the degree to which reduced functional capacity limited the patient's work ability was one of the most problematic issues for both groups, with more than half of the OSs and 80 % of the GPs perceiving this as problematic. A majority of the GPs also found it problematic to ascertain optimal time and degree of sickness certification. There is, so far, no scientific evidence on the best or optimal sick-listing of patients which makes this a difficult task for many physicians [1]. In order to assess the patient's work ability, the physician also needs information about the patient's work place, and this information is difficult for physicians to obtain and validate [16]. Physicians often fail to contribute information needed by the SIO concerning functional capacity when they issue sickness certificates [1,30].

Handling situations in which the physician and patient have different opinion about the need for sickness absence and prolongation of sick-leave spells previously certified by another physician were themes reported as problematic by more than half of both groups. Writing the sickness certificates was the only issue not considered to be more problematic by GPs than by OSs. As most GPs recently had had special training from the SIO, one could have anticipated their rates to be even lower.

### Waiting times and prolonged sickness certification

A higher proportion of OSs than GPs reported issuing prolonged certifications every week due to waiting times in health care for medical examination, investigation, treatment or rehabilitation. GPs on the other hand more frequently issued prolonged certifications when treatment possibilities were lacking (e.g. lack of cognitive behavioural therapists) and when the patient was waiting for measures to be taken by the SIO, the employment office or the employer. These differences are possibly due to the fact that GPs more often need to cooperate with other stakeholders both within and outside the healthcare. GPs are thereby more dependent on others, while OSs might by themselves influence the waiting times influencing there work with patients.

### Organisational strategies

Overall, a higher rate of GPs reported more problems, as well as having support from management and common policies regarding sickness certification; they had more than two times higher odds of having such support and policies. Among the physicians reporting problems every week, the ones working as GPs perceived somewhat less support and fewer reported common policies, but on the other hand somewhat more of the OSs reported support from management. We did not ask the physicians if they experienced the support or the policies as helpful or not, so it is not possible to draw any conclusions about the pro or cons of support or common policies based on the results from this study.

### Implications and further research

As was shown in this study as in others physicians experience problems related to their sick-listing task. Physicians perceive problems due to where and with what they work. In order to plan for educational activities and interventions in order to improve the management of sickness certification, we need knowledge about experiences of different groups of physicians. This study has helped us to meet the different need of vocational training for GPs and OSs. Further research, with other types of study design, taking into account organisational differences, different patient-mixes, the possibility of referring problematic patients, and other factors that might be of importance is, however, needed to explain the differences in sick-listing issues between physician groups. We also need more studies in order to settle best practice and how to organise health care services appropriately regarding sick-listing, especially regarding patients with ambiguous diagnoses.

## Conclusion

Both GPs and OSs frequently experience a variety of problems with sickness certification, with GPs predominating regarding most personal stressors and clinical problems. The personal strategies to handle the problems differed widely between the two groups. The explanation to the differences regarding the severity of perceived problems and possibilities of coping with sickness certification problems might be due to contextual differences, different patient-mixes, the possibility of referring problematic patients and organisational strategies.

## Abbreviations

CI confidence interval

GP general practitioner

OR crude odds ratio

OS orthopaedic surgeon

SIO Social Insurance Office

## Competing interests

The author(s) declare that they have no competing interests.

## Authors' contributions

All authors have made substantial contribution to the study and have given final approval of the version to be published. BA, KA and SP were responsible for the conception and design of the study. BA, JH and SP made the data analysis and all authors were involved in the interpretation of data. BA, GN and SP drafted the manuscript and the others revised it critically.

## Pre-publication history

The pre-publication history for this paper can be accessed here:


